# Fetal Growth and Risk of Stillbirth: A Population-Based Case–Control Study

**DOI:** 10.1371/journal.pmed.1001633

**Published:** 2014-04-22

**Authors:** Radek Bukowski, Nellie I. Hansen, Marian Willinger, Uma M. Reddy, Corette B. Parker, Halit Pinar, Robert M. Silver, Donald J. Dudley, Barbara J. Stoll, George R. Saade, Matthew A. Koch, Carol J. Rowland Hogue, Michael W. Varner, Deborah L. Conway, Donald Coustan, Robert L. Goldenberg, for the Eunice Kennedy Shriver National Institute of Child Health and Human Development Stillbirth Collaborative Research Network

**Affiliations:** 1 University of Texas Medical Branch at Galveston, United States of America; 2 RTI International, Research Triangle Park, North Carolina, United States of America; 3 The Pregnancy and Perinatology Branch, the Eunice Kennedy Shriver National Institute of Child Health and Human Development, National Institutes of Health, Bethesda, Maryland, United States of America; 4 Brown University School of Medicine, Providence, Rhode Island, United States of America; 5 University of Utah School of Medicine and Intermountain Health Care, Salt Lake City, Utah, United States of America; 6 University of Texas Health Science Center at San Antonio, United States of America; 7 Emory University School of Medicine and Children's Healthcare of Atlanta, Atlanta, Georgia, United States of America; 8 Rollins School of Public Health, Emory University, Atlanta, Georgia, United States of America; 9 Columbia University Medical Center, New York, New York, United States of America; University of Queensland, Australia

## Abstract

**Background:**

Stillbirth is strongly related to impaired fetal growth. However, the relationship between fetal growth and stillbirth is difficult to determine because of uncertainty in the timing of death and confounding characteristics affecting normal fetal growth.

**Methods and Findings:**

We conducted a population-based case–control study of all stillbirths and a representative sample of live births in 59 hospitals in five geographic areas in the US. Fetal growth abnormalities were categorized as small for gestational age (SGA) (<10th percentile) or large for gestational age (LGA) (>90th percentile) at death (stillbirth) or delivery (live birth) using population, ultrasound, and individualized norms. Gestational age at death was determined using an algorithm that considered the time-of-death interval, postmortem examination, and reliability of the gestational age estimate. Data were weighted to account for the sampling design and differential participation rates in various subgroups. Among 527 singleton stillbirths and 1,821 singleton live births studied, stillbirth was associated with SGA based on population, ultrasound, and individualized norms (odds ratio [OR] [95% CI]: 3.0 [2.2 to 4.0]; 4.7 [3.7 to 5.9]; 4.6 [3.6 to 5.9], respectively). LGA was also associated with increased risk of stillbirth using ultrasound and individualized norms (OR [95% CI]: 3.5 [2.4 to 5.0]; 2.3 [1.7 to 3.1], respectively), but not population norms (OR [95% CI]: 0.6 [0.4 to 1.0]). The associations were stronger with more severe SGA and LGA (<5th and >95th percentile). Analyses adjusted for stillbirth risk factors, subset analyses excluding potential confounders, and analyses in preterm and term pregnancies showed similar patterns of association. In this study 70% of cases and 63% of controls agreed to participate. Analysis weights accounted for differences between consenting and non-consenting women. Some of the characteristics used for individualized fetal growth estimates were missing and were replaced with reference values. However, a sensitivity analysis using individualized norms based on the subset of stillbirths and live births with non-missing variables showed similar findings.

**Conclusions:**

Stillbirth is associated with both growth restriction and excessive fetal growth. These findings suggest that, contrary to current practices and recommendations, stillbirth prevention strategies should focus on both severe SGA and severe LGA pregnancies.

*Please see later in the article for the Editors' Summary*

## Abstract 2

Radek Bukowski and colleagues conducted a case control study in 59 US hospitals to determine the relationship between fetal growth and stillbirth, and find that both restrictive and excessive growth could play a role.

*Please see later in the article for the Editors' Summary*

## Editors' Summary

**Background:**

Pregnancy is usually a happy time, when the parents-to-be anticipate the arrival of a new baby. But, sadly, about 20% of pregnancies end in miscarriage—the early loss of a fetus (developing baby) that is unable to survive independently. Other pregnancies end in stillbirth—fetal death after 20 weeks of pregnancy (in the US; after 24 weeks in the UK). Stillbirths, like miscarriages, are common. In the US, for example, one in every 160 pregnancies ends in stillbirth. How women discover that their unborn baby has died varies. Some women simply know something is wrong and go to hospital to have their fears confirmed. Others find out when a routine check-up detects no fetal heartbeat. Most women give birth naturally after their baby has died, but if the mother's health is at risk, labor may be induced. Common causes of stillbirth include birth defects and infections. Risk factors for stillbirth include being overweight and smoking during pregnancy.

**Why Was This Study Done?:**

Stillbirths are often associated with having a “small for gestational age” (SGA) fetus. Gestation is the period during which a baby develops in its mother's womb. Gestational age is estimated from the date of the woman's last menstrual period and/or from ultrasound scans. An SGA fetus is lighter than expected for its age based on observed distributions (norms) of fetal weights for gestational age. Although stillbirth is clearly associated with impaired fetal growth, the exact relationship between fetal growth and stillbirth remains unclear for two reasons. First, studies investigating this relationship have used gestational age at delivery rather than gestational age at death as an estimate of fetal age, which overestimates the gestational age of stillbirths and leads to errors in estimates of the proportions of SGA and “large for gestational age” (LGA) stillbirths. Second, many characteristics that affect normal fetal growth are also associated with the risk of stillbirth, and this has not been allowed for in previous studies. In this population-based case–control study, the researchers investigate the fetal growth abnormalities associated with stillbirth using a new approach to estimate gestational age and accounting for the effect of characteristics that affect both fetal growth and stillbirth. A population-based case–control study compares the characteristics of patients with a condition in a population with those of unaffected people in the same population.

**What Did the Researchers Do and Find?:**

The researchers investigated all the stillbirths and a sample of live births that occurred over 2.5 years at 59 hospitals in five US regions. They used a formula developed by the Stillbirth Collaborative Research Network to calculate the gestational age at death of the stillbirths. They categorized fetuses as SGA if they had a weight for gestational age within the bottom 10% (below the 10th percentile) of the population and as LGA if they had a weight for gestational age above the 90th percentile at death (stillbirth) or delivery (live birth) using population, ultrasound, and individualized norms of fetal weight for gestational age. Population norms incorporate weights for gestational age from normal pregnancies and from pregnancies complicated by growth abnormalities, whereas the other two norms include weights for gestational age from normal pregnancies only. Having an SGA fetus was associated with a 3- to 4-fold increased risk of stillbirth compared to having a fetus with “appropriate” weight for gestational age based on all three norms. LGA was associated with an increased risk of stillbirth based on the ultrasound and individualized norms but not the population norms. Being more severely SGA or LGA (below the 5th percentile or above the 95th percentile) was associated with an increased risk of stillbirth.

**What Do These Findings Mean?:**

These findings indicate that, when the time of death is accounted for and norms for weight for gestational age only from uncomplicated pregnancies are used, stillbirth is associated with both restricted and excessive fetal growth. Overall, abnormal fetal growth was identified in 25% of stillbirths using population norms and in about 50% of stillbirths using ultrasound or individualized norms. Although the accuracy of these findings is likely to be affected by aspects of the study design, these findings suggest that, contrary to current practices, strategies designed to prevent stillbirth should focus on identifying both severely SGA and severely LGA fetuses and should use norms for the calculation of weight for gestational age based on normal pregnancies only. Such an approach has the potential to identify almost half of the pregnancies likely to result in stillbirth.

**Additional Information:**

Please access these websites via the online version of this summary at http://dx.doi.org/10.1371/journal.pmed.1001633.

- The March of Dimes, a nonprofit organization for pregnancy and baby health, provides information on stillbirth
- Tommy's, a UK nonprofit organization that funds research into stillbirth, premature birth, and miscarriage and provides information for parents-to-be, also provides information on stillbirth (including personal stories)
- The UK National Health Service Choices website provides information about stillbirth (including a video about dealing with grief after a stillbirth)
- MedlinePlus provides links to other resources about stillbirth (in English and Spanish)
- Information about the Stillbirth Collaborative Research Network is available

## Introduction

One in 160 births at ≥20 wk gestation in the United States is stillborn, resulting in over 25,000 stillbirths each year [1], a rate similar to the rate of infant death [2]. Stillbirth also constitutes one of the main causes of mortality worldwide [3],[4]. Fetal growth restriction, and its proxy small for gestational age (SGA) fetus, is one of the most important predictors of stillbirth because of its strong association with stillbirth and relatively high prevalence. Fetal growth restriction is a pathological decrease in the rate of fetal growth that frequently results in an adverse outcome, but is difficult to define because it requires serial evaluation of fetal growth. Conversely, SGA is easier to define—a birth weight smaller than expected—but includes a proportion of small but normal pregnancies. The overlap between fetal growth restriction and SGA depends on the norm and cutoff used. Approximately a quarter of stillbirths are associated with SGA fetus, almost double the proportion associated with any other risk factor [5],[6]. For these reasons, standards of clinical practice recommend evaluation of fetal growth during each prenatal visit and further evaluation and possible intervention if the fetal growth rate is poor [7]–[10]. Despite ubiquitous use of fetal growth monitoring in clinical practice and extensive research, the relationship between fetal growth and stillbirth is not completely understood, for the following reasons.

First, birth weight percentile is a function of birth weight and age of the fetus. Thus, studies using gestational age (GA) at delivery rather than GA at death, as an estimate of the age of the fetus, systematically overestimate the GA of stillbirths. This leads to overestimation of the proportion of birth weights considered SGA and underestimation of birth weights considered large for GA (LGA) among stillbirths.

Second, a number of maternal characteristics such as weight, height, age, race/ethnicity, parity, exposures during pregnancy, smoking, and measures of placental function (such as maternal blood concentrations of placental hormones) have been observed to influence the magnitude of both fetal growth and risk of stillbirth [5],[6],[11]–[14]. Specific ranges of these characteristics are also associated with birth weight among uncomplicated live birth pregnancies and are accounted for by using individualized birth weight norms [15]. Thus, to accurately assess fetal growth and identify fetal growth abnormalities, an accurate estimate of GA and accounting for the effect of physiologic determinants of fetal growth are needed. This is especially important in stillbirths, because their exact GA is mostly not known, and GA at delivery overestimates GA of stillbirths. Moreover, many characteristics that affect the growth of live and normal pregnancies, such as maternal race/ethnicity, body mass index (BMI), and parity, are also associated with the risk of stillbirth. The effect of these characteristics on fetal growth and risk of stillbirth is complex, because within a certain range their effect on fetal growth is observed among normal live pregnancies without complications [15].

Therefore, we hypothesized that accounting for time of death in determining fetal age and birth weight percentile, and determining percentiles of birth weight based on norms that account for factors affecting birth weight in normal pregnancies, will more accurately identify the proportion of stillbirths associated with abnormal growth than using traditional methods.

## Methods

### Ethics Statement

This study was approved by the institutional review boards at each of the clinical recruiting sites and at the data coordinating center. All mothers participating in the study gave written informed consent.

### Study Design

The Stillbirth Collaborative Research Network (SCRN) conducted a population-based case–control study of stillbirths in the United States. The study design has been previously described [16]. Briefly, the population consisted of residents of five geographic areas defined a priori by state and county lines: (1) the state of Rhode Island and Bristol County, Massachusetts; (2) DeKalb County, Georgia; (3) Galveston and Brazoria Counties, Texas; (4) Bexar County, Texas; and (5) Salt Lake County, Utah. Participants were recruited at delivery between March 1, 2006, and September 30, 2008, from 59 community and academic, urban, and rural hospitals in the five areas, with a cumulative average of 80,000 deliveries per year. The investigators selected the 59 hospitals to ensure access to at least 90% of all pregnancies ending in either stillbirth or live birth within each geographic area based on estimates of the number of hospital deliveries from vital statistics data and hospital medical records available during study planning. All women whose pregnancies resulted in stillbirth and a representative sample of women with live births, oversampled for those delivering at <32 wk gestation and those of African descent delivering at ≥32 wk gestation, were approached for enrollment. Terminations of pregnancy were excluded.

A stillbirth was defined by Apgar scores of 0 at 1 and 5 min, and no signs of life by direct observation. The protocol included an in-hospital maternal interview, medical record abstraction, placental pathology examination, and biospecimen collection for stillbirths and live births. For stillbirths, a standardized postmortem examination was also performed.

### Fetal Age

Fetal age was based on an estimated due date and the date of delivery for live births and an estimated due date and estimated date of death for stillbirths, using an algorithm developed by the SCRN investigators [17]. For both stillbirths and live births, the due date was estimated using (1) menstrual dating criteria that agreed with ultrasound or ultrasound dating criteria (when the last menstrual period date was unknown or uncertain, or menstrual dating did not agree with ultrasound dating within specified constraints) in 88.3% of pregnancies, (2) menstrual dating alone when ultrasound was not available in 6.1%, and (3) review of all clinical information available in 5.6%.

The SCRN algorithm for estimating time of death and fetal age at death in stillbirths considered the following: the reliability of the estimated due date; the length of the interval between the time the fetus was last documented alive and the time fetal demise was first recorded based on information from prenatal care visits, hospitalizations, and ultrasound examinations (the time-of-death interval); and information available from postmortem examination, including degree of fetal maceration and foot length measurement. The estimated due date was considered reliable if estimated by ultrasound or by certain menstrual dating that agreed with ultrasound (within specified constraints). Briefly, (1) if the estimated due date was reliable, the fetal age at death was estimated using the due date and the date fetal demise was diagnosed (if the interval during which death occurred was ≤1 d) or the date at the midpoint of the time-of-death interval (if the interval was >1 and ≤7 d); (2) if the estimated due date was unreliable, or the time-of-death interval was >7 d, GA at death was estimated using foot length, or using GA reported by the study site at screening based on clinical criteria when foot length information was not available. Precise estimates of GA at death, defined as meeting reliable dating criteria and having an interval of 1 wk or less during which the demise could have occurred, were possible for 47% of stillbirths.

The fetal age at death estimated by the SCRN algorithm was used for all stillbirths in the primary analysis. For comparison, GA at stillbirth delivery, calculated using the estimated due date and the delivery date, and GA at delivery minus 2 d were also used as estimates of fetal age at death to examine the impact on birth weight percentiles. For live births, fetal age at delivery was estimated using the estimated due date if reliable and the delivery date, or if the estimated due date was unreliable, the GA reported by the study site at screening was used.

### Fetal Weight

**Figure 1:**
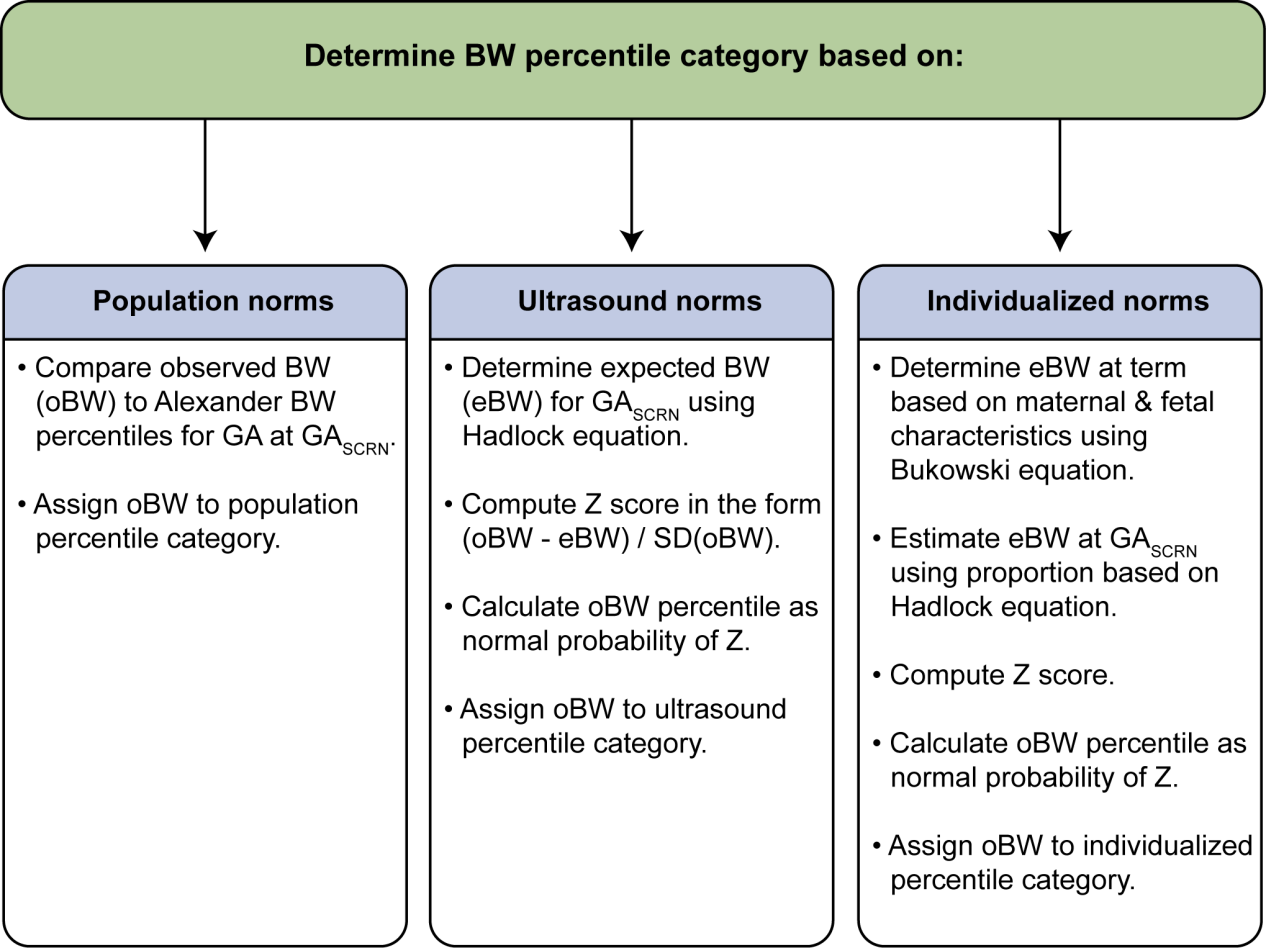
Summary of steps used to assign infants to a birth weight percentile category. GA_SCRN_ is the fetal age at death (for stillbirths) or delivery (for live births) estimated by the SCRN algorithm [17]. BW, birth weight; SD, standard deviation. References: Alexander et al. [18], Hadlock et al. [19]; Bukowski et al. [15].

Fetal weight percentiles were determined based on birth weight and the fetal age estimate for stillbirths and live births. Birth weight was obtained from medical records or postmortem examination and was compared to expected weight for GA based on three types of norms: population, ultrasound, and individualized norms [15],[18],[19]. Alexander et al.'s population norms reported percentiles of birth weight for completed weeks of GA, 20–44 wk, based on data from the population of over 3 million US single live births in 1991 [18]. SCRN live birth and stillbirth birth weights were compared to Alexander et al.'s population norms to determine a percentile category. Hadlock et al.'s ultrasound norms used fetal weight estimated in utero by ultrasound in 392 uncomplicated pregnancies progressing to term to develop an equation to predict fetal weight as a function of GA [19]. The observed SCRN birth weight was compared to the fetal weight predicted by Hadlock et al.'s equation, with a percentile computed under normality assumptions. Bukowski et al.'s individualized norms used data from 9,818 uncomplicated pregnancies resulting in singleton births to develop an equation to predict expected term birth weight based on a number of maternal and fetal characteristics affecting normal fetal growth [15]. A proportion derived using Hadlock et al.'s equation [19] was then used to adjust the prediction at term to a prediction at the GA of the SCRN live birth or stillbirth, and the observed birth weight was then compared to this individualized predicted birth weight, with a percentile computed under normality assumptions. Steps used to estimate percentiles are summarized in Figure 1, and details of estimation are provided for each norm below. SGA birth weight was defined as birth weight less than the 10th percentile, and LGA as birth weight above the 90th percentile for GA, while severe SGA and LGA were defined by the 5th and 95th percentiles respectively. Birth weights in the 10th–90th percentile range were classified as appropriate for age (AGA).

#### Population norms

The estimated GA at death (for stillbirths) or delivery (for live births) using the SCRN algorithm (GA_SCRN_) was recorded in weeks, with days retained as a fractional component. Alexander et al. reported 5th, 10th, 50th, 90th, and 95th percentiles of birth weight for completed weeks of GA derived using data from over 3 million US 1991 single live births [18]. Linear interpolation was used with Alexander et al.'s reported percentiles for each completed week of GA, 20–44 wk, to derive birth weight percentiles for GA_SCRN_. For interpolation, each percentile reported by Alexander et al. was taken to represent birth weight at the midpoint of the GA week range (0–6 d). For example, the 10th percentile of birth weight reported for an infant of 30 wk GA was considered the 10th percentile for 30.5 wk GA.

The observed birth weight of each infant was compared to the interpolated 5th, 10th, 50th, 90th, and 95th percentiles of birth weight for GA_SCRN_ and assigned to a percentile category (e.g., <10th percentile). Three infants with GA_SCRN_>44.5 wk were assigned to the 10th–90th percentile category based on birth weight between the 10th and 90th percentiles reported for 44 wk.

#### Ultrasound norms

Hadlock et al. used in utero fetal weight, estimated with ultrasound measurements, from 392 uncomplicated pregnancies progressing to term among predominantly middle-class white women to develop a best-fitting equation for fetal weight as a function of GA [19]. Furthermore, they estimated fetal weight percentiles by GA, assuming a Gaussian distribution for fetal weight on the natural log scale. In like manner, a birth weight percentile for GA_SCRN_ was estimated for each infant in the SCRN cohort as the normal probability of a *Z*-score, *Z*_ULTRASOUND_, calculated as follows:
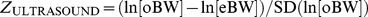
(1)where ln(oBW) is the natural log of the observed birth weight of the SCRN infant, SD(ln[oBW]) is the standard deviation of birth weight on the natural log scale reported by Hadlock et al. ( = 0.12), and ln(eBW) is the natural log of the expected birth weight for GA_SCRN_ by Hadlock et al.'s equation:
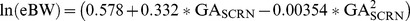
(2)


#### Individualized norms

Bukowski et al. developed a regression model for birth weight in a normal population using singleton births from 9,818 women with uncomplicated pregnancies [15]. The model equation accounts for maternal and other characteristics affecting birth weight (see Table 2 of [15]). This equation, with some modifications, was used to compute an individualized expected birth weight at 280 d for each SCRN infant. The modifications were as follows. (1) Maternal weight in the first trimester was not available, and maternal pre-pregnancy weight was substituted. (2) Fetal heart rate in the first trimester was not available and could not be included. (3) GA was not needed in the prediction equation when estimating birth weight at 280 d, as Bukowski et al.'s regression model used GA minus 280. (4) SCRN marital status categories were “not married,” “cohabitating,” and “married.” For the purposes of modeling, “not married” was given the coefficient for “single,” and “cohabitating” was given the coefficient for “other” in Table 2 of [15]. (5) Values for continuous variables outside the range of values observed in the population in which the model equation was developed were truncated to conform to those ranges. Values were truncated for nine of the 19 variables included in the model equation. For eight of these nine variables—maternal weight, cigarettes/day, nuchal translucency size, and maternal blood concentrations of five placental hormones—values were truncated for between 1 and 21 infants (representing <1% to 3.8%). DeltaGA (the difference between GA estimated by first trimester ultrasound and age estimated by last menstrual period date) was truncated for 190 infants with measures outside the −7 to 7 range of the data included in the modeling (representing 29%). (6) Accommodations were made for a number of variables that were missing. Missing values for ordinal variables, with the exception of education, were set to mean or median values (maternal weight: 64 kg; maternal height: 165 cm; number of prior term pregnancies: 0; number of prior abortions: 0; number of cigarettes/day: 0; first and second trimester test results in multiples of the median: 1; DeltaGA: 0). For each categorical variable, and for education, the coefficient for “missing” values was derived as a weighted average of the coefficients of the non-missing levels, with weights taken as the proportion of the corresponding level in the SCRN singleton cohort.

**Table 2:** Variables used to compute individualized expected birth weight at 280(unweighted *n* = 2,348).

Variable	*N*	*N* Missing
Maternal weight	2,293	55
Maternal height	2,334	14
Race/ethnicity	2,347	1
Maternal education	2,230	118
Marital status	2,238	110
Number of prior term pregnancies	2,345	3
Number of prior abortions	2,347	1
Altitude of residence	2,348	0
Cigarettes/day first trimester	2,236	112
Ovulation induction	2,346	2
Nuchal translucency size	76	2,272
Pregnancy-associated plasma protein A	103	2,245
Free beta human chorionic gonadotropin	42	2,306
Alpha-fetoprotein	870	1,478
Inhibin A	576	1,772
Total human chorionic gonadotropin	838	1,510
Unconjugated estriol	831	1,517
First trimester size (DeltaGA)	621	1,727
Male fetus	2,341	7

After computing an individualized expected birth weight at 280 d (BW_INDIV280_) for each SCRN infant, an individualized expected birth weight for GA_SCRN_ (eBW) was calculated using a proportion derived using Hadlock et al.'s equation [19]:
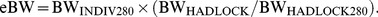
(3)where BW_HADLOCK_ is the predicted birth weight for GA_SCRN_ using Hadlock et al.'s equation and BW_HADLOCK280_ is the predicted birth weight for GA at 280 d using Hadlock et al.'s equation ( = 3,619.17 g).

Finally, an individualized birth weight percentile was estimated as the normal probability of an individualized *Z*-score, *Z*_INDIV_, of the form:
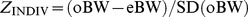
(4)where oBW is the observed birth weight of the SCRN infant, eBW is the individualized expected birth weight at GA_SCRN_, and SD(oBW) is an estimate of the standard deviation of the observed birth weight. The standard deviation was estimated using an estimate of the coefficient of variation at 280 d (CV_INDIV280_) multiplied by eBW (CV_INDIV280_×eBW). CV_INDIV280_ was taken as the reported square root of the residual mean square from the model from Bukowski et al. (358.479 g) divided by the expected birth weight at 280 d from Hadlock et al. (BW_HADLOCK280_ = 3,619.17 g). Thus, CV_INDIV280_ = 358.479/3,619.17.

In sensitivity analyses, individualized norm percentiles were recalculated based on three different reduced prediction equations that included subsets of variables largely non-missing in the SCRN cohort, instead of the original 19 variables. First, percentiles were estimated based on a prediction equation that included the 11 variables with largely non-missing values in the SCRN cohort (maternal weight, height, race/ethnicity, education, marital status, number of prior term pregnancies, number of prior abortions, altitude of residence, use of ovulation induction to become pregnant, cigarettes smoked per day during the first trimester, and male fetus), with coefficients derived using data from the original Bukowski et al. population. Second, percentiles were estimated based on a prediction equation that included the six variables suggested previously [20] (maternal weight, height, race/ethnicity, number of prior term pregnancies, cigarettes smoked per day during the first trimester, and male fetus), with coefficients derived using data from the original Bukowski et al. population. Third, percentiles were estimated based on a prediction equation that included five of the six variables listed previously (cigarette smoking was excluded), with coefficients derived using data from the original Bukowski et al. population.

### Statistical Analysis

Data were weighted for the analysis. The analysis weights were constructed in steps. First, weights were constructed that took into account the sampling design, including staggered enrollment starts across the 59 site hospitals, and different sampling probabilities associated with the oversampling of live births <32 wk gestation and live births at ≥32 wk gestation to women of African descent. Some women screened and determined eligible for the study were not approached, and others were approached but did not consent. Additional weight adjustments were constructed to account for this nonresponse and utilized information collected at screening to determine characteristics associated with the likelihood of participating. The final analysis weights were constructed as the product of these weighting factors. Details of the live birth sample selection procedure and construction of the analysis weights have been reported previously [16]. The weighted sample was intended to approximate a random selection of live births in the five geographic areas during the enrollment period, with proportions according to GA and maternal race reflective of the population, and to reduce potential bias associated with failing to include all women eligible for participation.

The analysis was restricted to singleton stillbirths and live births with non-missing birth weight and GA≥20 wk at death or delivery. Statistical significance for comparisons of characteristics between stillbirths and live births was determined by the median or chi-square test, and for comparisons between proportions among stillbirths by the McNemar test. Crude and adjusted odds ratios (ORs) and 95% confidence intervals were calculated from logistic regression models. Primary results used all stillbirths and all live births. Term stillbirth and live birth were defined as GA of 37 wk 0 d or more. To assess the association of SGA and LGA with preterm stillbirth, the logistic model was restricted to preterm stillbirth versus all live births. Multivariable models used to estimate adjusted ORs included study site (five geographic areas) and stillbirth risk factors known at the beginning of pregnancy [21]. Approximately 10% of observations were missing values of covariates included in the multivariable models and were omitted from computations of adjusted ORs. Weighted analyses were conducted using SUDAAN software version 10.0.1.

## Results

**Figure 2:**
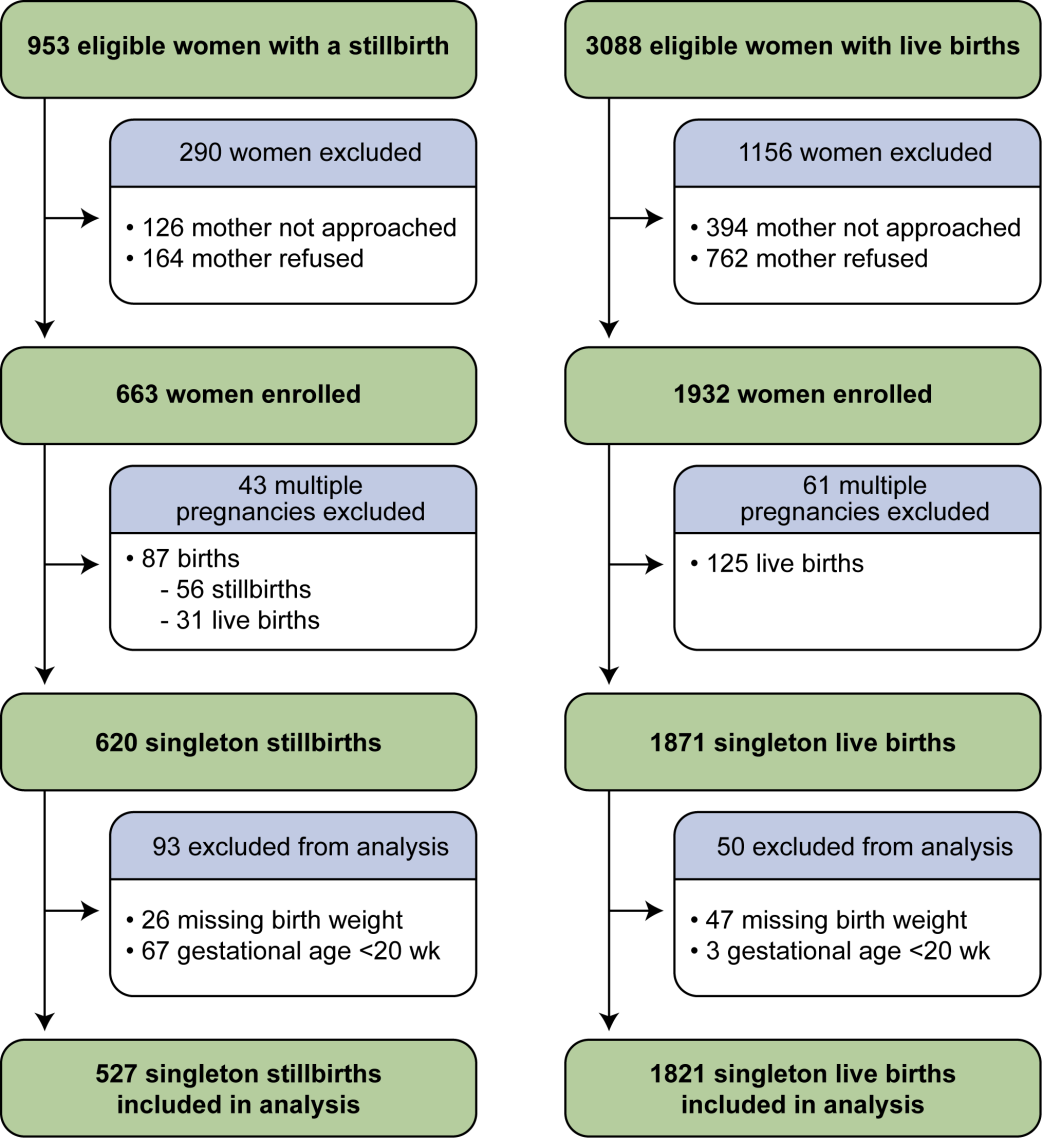
Study enrollment and inclusion in analysis.

The SCRN identified 953 women with stillbirths and 3,088 women with live births eligible for participation in the study. Of these, 663 (70%) women with stillbirths and 1,932 (63%) women with live births consented to participate. Women with stillbirths enrolled in the study did not differ from those not enrolled on maternal age, maternal race/ethnicity, insurance/method of payment, or GA at delivery [21]. Women with live births enrolled in the study did not differ from those not enrolled on maternal age and insurance/method of payment. However, the proportion of Hispanic women was larger among those enrolled than among those not enrolled (34% versus 27%), and the proportions of non-Hispanic black and non-Hispanic white women were smaller (21% versus 25% and 41% versus 42%, respectively). Enrolled and non-enrolled women with live births also differed on GA at delivery, with a larger proportion of births ≥37 wk among those enrolled (75% versus 69%) [21]. From the pregnancies of women enrolled, 527 singleton stillbirths and 1,821 singleton live births were included in these analyses (Figure 2).

Women who delivered stillbirths were more likely than women who delivered live births to be non-Hispanic black (23% versus 11%, *p*<0.001), overweight (BMI≥25 kg/m^2^) (57% versus 46%, *p*<0.01), single (52% versus 39%, *p*<0.001), smokers before pregnancy (21% versus 14%, *p*<0.01), and less educated (less than 12 y of school) (24% versus 19%, *p*<0.001). They were more likely to have hypertension (11% versus 5%, *p*<0.001), to have pregestational diabetes (5% versus 2%, *p*<0.001), and to be nulliparous or to have had a prior stillbirth (*p*<0.001) (Table 1). As expected, stillborn infants had lower GA (median 28 versus 39 wk, *p*<0.001) and lower birth weight (*p*<0.001) and were more likely to have congenital malformations than live born infants (13% versus 3%, *p*<0.001) (Table 1).

**Table 1:** Characteristics of stillbirth and live birth pregnancies.

Category	Characteristic^a^	Stillbirth	Live Birth	*p*-Value^b^
	**Weighted sample size, number** c	528	1,382	
**Mother**	**Maternal age at delivery, years**			0.50
	Median	26	26	
	Interquartile range	21 to 32	22 to 31	
	**Paternal age at delivery, years**			0.02
	Median	28	29	
	Interquartile range	23 to 34	24 to 34	
	**Maternal race/ethnicity, percent**			<0.001
	Non-Hispanic white	34	46	
	Non-Hispanic black	23	11	
	Hispanic	38	36	
	Other	6	8	
	**BMI, percent**			0.003
	<18.5	4	3	
	18.5–24.9	40	50	
	25–29.9	26	23	
	30–34	15	12	
	≥35	16	11	
	**Maternal education, grade, percent**			<0.001
	0–11	24	19	
	12	31	26	
	≥13	45	55	
	**Marital status, percent**			<0.001
	Not married	26	15	
	Cohabiting	26	24	
	Married	48	60	
	**Insurance, percent**			0.02
	No insurance	5	4	
	Any public/private assistance	55	49	
	VA/commercial health insurance/HMO	40	47	
	**Family income last 12 mo, percent**			0.13
	Only public/private assistance	9	6	
	Assistance and personal income	38	38	
	Only personal income	53	56	
	**Blood type, percent**			0.31
	A	31	34	
	B	14	11	
	O	51	51	
	AB	4	3	
	**Rh negative, percent**	9	11	0.20
	**Smoking status, percent** d			0.003
	Did not smoke	79	86	
	<10 cigarettes/day	11	7	
	≥10 cigarettes/day	10	7	
	**Alcohol consumption, percent** d			0.60
	Did not drink	58	58	
	Drank, no bingeing	21	23	
	Binged	21	19	
	**Lifetime drug use, percent**			0.03
	Never	66	69	
	Ever, without addiction	29	28	
	Ever, with addiction	5	2	
	**Medical history, percent**			
	Hypertension	11	5	<0.001
	Diabetes	5	2	<0.001
	Seizure disorder	3	2	0.23
	**Pregnancy history, percent**			<0.001
	Nulliparous; never pregnant or only elective terminations	36	30	
	Nulliparous with previous losses	11	5	
	Multiparous with no previous losses at <20 wk or stillbirths	31	46	
	Multiparous with no stillbirth but previous losses at <20 wk	14	17	
	Multiparous with stillbirth	7	2	
**Infant**	**GA, weeks** e			<0.001
	Median	28	39	
	Interquartile range	23 to 36	38 to 40	
	**GA category, percent**			<0.001
	20–23 wk	33	<1	
	24–27 wk	17	<1	
	28–31 wk	11	1	
	32–36 wk	21	9	
	37+ wk	18	89	
	**Birth weight, grams**			<0.001
	Median	992	3,317	
	Interquartile range	454 to 2,468	2,978 to 3,628	
	**Male sex, percent**	52	50	0.55
	**Congenital anomaly, percent**	13	3	<0.001

a Information was missing as follows (unweighted missing *n* for stillbirths and live births, respectively): paternal age (44 and 98), maternal race/ethnicity (1 stillbirth), BMI (21 and 42), maternal education (36 and 82), marital status (34 and 76), insurance (3 and 4), family income (40 and 90), blood type (5 and 6), Rh factor (2 and 6), cigarette smoking (34 and 78), alcohol consumption (37 and 81), drug use (38 and 91), hypertension (2 and 5), diabetes (1 and 5), seizure disorder (2 and 6), male sex (5 and 2).

Percentages may not sum to 100 because of rounding.

b *p*-Value for a difference between stillbirths and live births by the median test (continuous variables) or the Wald chi-square test (categorical variables).

c Analysis weights that accounted for the basic study design plus other aspects of the sampling were used.

Unweighted sample sizes were 527 stillbirths and 1,821 live births.

d Average number of cigarettes smoked per day during the 3 mo prior to pregnancy or alcohol consumption during the 3 mo prior to pregnancy.

Drank without bingeing was defined as 0–6 drinks in a typical week and no occasion where four or more drinks were consumed in a single time period (“binge”). Bingeing was defined as at least one binge and/or seven or more drinks in a typical week.

e GA at death (stillbirths) or delivery (live births) by the SCRN algorithm [17].

HMO, health maintenance organization; VA, Veterans Affairs.

Some variables used to compute individualized norms were missing in a proportion of pregnancies and were set to mean, median, or weighted average (Table 2). However, in sensitivity analyses, individualized norm percentiles derived using a subset of 11 variables largely non-missing in the cohort, or derived from a subset of six or five variables suggested previously [20] (with or without smoking), yielded patterns of association with stillbirth similar to those derived using the full model (Table 3). Thus, accounting for maternal smoking in computing individualized norms did not affect the association between fetal growth abnormalities and stillbirth. The five-variable model without smoking also showed that the association between SGA and LGA and risk of stillbirth was observed when individualized norms were derived without including factors with potentially adverse effects on pregnancy. However, it could be argued that smoking should not be used in individualization of fetal growth because of its potential adverse effect on a given pregnancy.

**Table 3:** Birth weight percentiles among stillbirths and live births using different equations to estimate individualized expected weight.

Birth Weight Norms and Percentiles	SB	LB	Crude OR for SB (95% CI)^a^	Adjusted OR for SB (95% CI)^b^
**Weighted sample size, number** c	528	1,382		
**Individualized norms, percent** d **(original 19 variables)**				
<5th percentile	33	9	6.47 (4.91–8.53)	6.01 (4.41–8.20)
5th–<10th	8	6	2.05 (1.35–3.12)	1.84 (1.13–2.98)
10th–90th	42	72	Reference	Reference
>90th–95th	4	5	1.39 (0.85–2.26)	1.48 (0.87–2.52)
>95th	13	7	3.04 (2.14–4.30)	2.57 (1.73–3.81)
**Individualized norms, percent** e **(11 variables)**				
<5th percentile	33	10	5.82 (4.38–7.71)	5.33 (3.92–7.26)
5th–<10th	8	6	2.33 (1.55–3.50)	2.07 (1.29–3.32)
10th–90th	43	73	Reference	Reference
>90th–95th	3	4	1.33 (0.78–2.25)	1.27 (0.69–2.31)
>95th	12	7	2.67 (1.88–3.80)	2.21 (1.49–3.28)
**Individualized norms, percent** f **(6 variables)**				
<5th percentile	30	7	6.80 (5.08–9.12)	6.24 (4.49–8.67)
5th–<10th	8	5	2.33 (1.48–3.65)	2.06 (1.26–3.35)
10th–90th	43	72	Reference	Reference
>90th–95th	4	6	1.17 (0.73–1.89)	1.45 (0.83–2.54)
>95th	15	9	2.58 (1.87–3.56)	2.21 (1.54–3.17)
**Individualized norms, percent** g **(5 variables)**				
<5th percentile	30	7	6.64 (4.96–8.88)	6.25 (4.51–8.67)
5th–<10th	7	6	2.04 (1.30–3.20)	1.74 (1.06–2.84)
10th–90th	44	71	Reference	Reference
>90th–95th	4	7	0.95 (0.58–1.57)	1.04 (0.58–1.87)
>95th	14	9	2.66 (1.93–3.68)	2.39 (1.67–3.44)

Birth weight for GA at death (stillbirths) or delivery (live births) by the SCRN algorithm [17]. Percentages may add to slightly more or less than 100% because of rounding.

a Unadjusted OR for stillbirth for infants with birth weight in the percentile group shown compared to infants in the reference group from a logistic regression model that included effects for percentile group only.

b Adjusted OR for stillbirth for infants with birth weight in the percentile group shown compared to infants in the reference group from a logistic regression model that in addition to the percentile group indicators included study site number; paternal age (<20, 20–34, 35–39, ≥40 y); the following maternal variables (categorized as shown in Table 1 or as noted): maternal age (<20, 20–34, 35–39, ≥40 y), race/ethnicity, education, marital status, insurance/method of payment, family income, smoking during the 3 mo prior to pregnancy, alcohol use during the 3 mo prior to pregnancy, drug use, BMI, blood type, Rh factor, pregestational hypertension, pregestational diabetes, seizure disorder, and pregnancy history; and infant sex.

c Analysis weights that accounted for the basic study design plus other aspects of the sampling were used.

Unweighted sample sizes were 527 stillbirths and 1,821 live births. Unweighted (weighted) sample sizes included in computation of adjusted ORs were 452 (451) stillbirths and 1,665 (1,261) live births.

d Individualized norm percentiles were derived using the fetal weight equation from Bukowski et al. [15].

All 19 variables were used in the fetal equation here. (Fetal heart rate was included in the original Bukowski equation but was not collected by the SCRN study. GA in days minus 280 d drops out of the equation when predicting birth weight at 280 d.)

e Individualized norm percentiles were derived using the subset of 11 variables largely non-missing in the SCRN cohort in the fetal weight equation to predict term birth weight: maternal weight, height, race/ethnicity, education, marital status, number of prior term pregnancies, number of prior abortions, altitude of residence, use of ovulation induction to become pregnant, cigarettes smoked per day during the first trimester, and male fetus.

f Individualized norm percentiles were derived using the subset of six variables suggested by Gardosi et al. [20] in the fetal weight equation to predict term birth weight (excluding GA, which drops out): maternal weight, height, race/ethnicity, number of prior term pregnancies, cigarettes smoked per day during the first trimester, and male fetus.

g Individualized norm percentiles were derived using a subset of five variables (the six variables above minus number of cigarettes smoked) in the fetal weight equation to predict term birth weight: maternal weight, height, race/ethnicity, number of prior term pregnancies, and male fetus.

LB, live birth; SB, stillbirth.

SGA pregnancies were associated with a statistically significant 3- to 4-fold increased risk of stillbirth compared to AGA pregnancies using percentiles based on population, ultrasound, and individualized norms (OR [95% CI]: 3.0 [2.2 to 4.0]; 4.7 [3.7 to 5.9]; 4.6 [3.6 to 5.9], respectively). LGA birth weight was associated with a significantly increased risk of stillbirth using percentiles derived from the ultrasound and individualized norms (OR [95% CI]: 3.5 [2.4 to 5.0]; 2.3 [1.7 to 3.1], respectively), but not using percentiles based on the population norms (OR [95% CI]: 0.6 [0.4 to 1.0]). Abnormal fetal growth (SGA or LGA) was identified in 25% of stillbirths using population norms and in twice as many stillbirths using ultrasound (57%, *p*<0.001) and individualized norms (58%, *p*<0.001). The association between stillbirth and SGA and LGA infants was mainly due to the high risk of stillbirth among infants with birth weights in the <5th and >95th percentiles and was not substantially changed when adjusted for stillbirth risk factors (Table 4).

**Table 4:** Birth weight percentiles among stillbirths and live births.

Birth Weight Norms and Percentiles	SB	LB	Crude OR for SB (95% CI)^a^	Adjusted OR for SB (95% CI)^b^
**Weighted sample size, number** c	528	1,382		
**Individualized norms, percent** d				
<5th percentile	33	9	6.47 (4.91–8.53)	6.01 (4.41–8.20)
5th–<10th	8	6	2.05 (1.35–3.12)	1.84 (1.13–2.98)
10th–90th	42	72	Reference	Reference
>90th–95th	4	5	1.39 (0.85–2.26)	1.48 (0.87–2.52)
>95th	13	7	3.04 (2.14–4.30)	2.57 (1.73–3.81)
<10th	41	15	4.59 (3.59–5.88)	4.39 (3.34–5.78)
>90th	17	12	2.33 (1.73–3.14)	2.13 (1.52–2.97)
**Ultrasound norms, percent** e				
<5th percentile	36	10	6.32 (4.86–8.22)	5.44 (4.03–7.33)
5th–<10th	9	7	2.30 (1.54–3.43)	2.33 (1.50–3.62)
10th–90th	43	77	Reference	Reference
>90th–95th	4	3	2.50 (1.43–4.37)	1.99 (1.04–3.81)
>95th	8	3	4.39 (2.79–6.89)	3.71 (2.23–6.16)
<10th	45	17	4.67 (3.69–5.92)	4.27 (3.27–5.59)
>90th	12	6	3.48 (2.42–5.01)	2.90 (1.92–4.37)
**Population norms, percent** f				
<5th percentile	12	4	3.49 (2.36–5.16)	3.05 (1.99–4.67)
5th–<10th	9	4	2.51 (1.64–3.84)	2.18 (1.31–3.65)
10th–90th	75	84	Reference	1.00 (1.00–1.00)
>90th–95th	2	4	0.48 (0.22–1.05)	0.55 (0.22–1.40)
>95th	3	4	0.75 (0.42–1.32)	0.70 (0.40–1.22)
<10th	20	8	3.00 (2.22–4.04)	2.62 (1.86–3.68)
>90th	4	8	0.63 (0.40–1.01)	0.64 (0.39–1.05)

Birth weight for GA at death (stillbirths) or delivery (live births) by the SCRN algorithm [17]. Percentages may add to slightly more or less than 100% because of rounding.

a Unadjusted OR for stillbirth for infants with birth weight in the percentile group shown compared to infants in the reference group from a logistic regression model that included effects for percentile group only.

b Adjusted OR for stillbirth for infants with birth weight in the percentile group shown compared to infants in the reference group from a logistic regression model that in addition to the percentile group indicators included study site number; paternal age (<20, 20–34, 35–39, ≥40 y); the following maternal variables (categorized as shown in Table 1 or as noted): maternal age (<20, 20–34, 35–39, ≥40 y), race/ethnicity, education, marital status, insurance, family income, smoking during the 3 mo prior to pregnancy, alcohol use during the 3 mo prior to pregnancy, drug use, BMI, blood type, Rh factor, pregestational hypertension, pregestational diabetes, seizure disorder, and pregnancy history; and infant sex.

c Analysis weights that accounted for the basic study design plus other aspects of the sampling were used.

Unweighted sample sizes were 527 stillbirths and 1,821 live births. Unweighted (weighted) sample sizes included in computation of adjusted ORs were 452 (451) stillbirths and 1,665 (1,261) live births.

d Individualized norm percentiles were derived using the fetal weight for GA equation from Bukowski et al. [15].

e Ultrasound norm percentiles were derived using the fetal weight for GA equation and standard error from Hadlock et al. [19].

f Alexander et al. population norm percentiles of birth weight for GA were used [18].

Simple linear interpolation was used with the Alexander birth weight percentiles reported for completed weeks of GA in whole weeks to derive birth weight percentiles for GA in weeks and days.

LB, live birth; SB, stillbirth.

SGA and LGA defined using the ultrasound and individualized norms were also associated with significantly increased risk of stillbirth in the subsets of pregnancies without pregestational diabetes, gestational diabetes, hypertension, or preeclampsia; non-anomalous births at more than 24 wk of gestation; and pregnancies with optimal estimates of GA and time of death (Table 5). Fetuses classified as SGA based on the population reference were also at significantly increased risk of stillbirth in these subsets, but those classified as LGA were not.

**Table 5:** Birth weight percentiles among subsets of stillbirths and live births.

Birth Weight Norms and Percentiles	Subset without Maternal Diabetes or Hypertension/Preeclampsia			Non-Anomalous Singletons ≥24 wk Gestation			Subset with Optimal Estimates of GA^a^		
	SB	LB	Crude OR for SB (95% CI)^b^	SB	LB	Crude OR for SB (95% CI)^b^	SB	LB	Crude OR for SB (95% CI)^b^
**Weighted sample size, number** c	382	1,079		310	1,339		200	972	
**Individualized norms, percent** d									
<5th percentile	30	8	6.14 (4.40–8.56)	36	8	7.50 (5.44–10.36)	28	8	5.50 (3.63–8.33)
5th–<10th	8	7	2.08 (1.30–3.34)	9	7	2.40 (1.47–3.92)	10	6	2.45 (1.36–4.43)
10th–90th	45	74	Reference	41	72	Reference	46	73	Reference
>90th–95th	5	5	1.46 (0.84–2.53)	3	5	1.12 (0.58–2.15)	4	6	1.00 (0.45–2.19)
>95th	13	7	3.15 (2.10–4.74)	10	7	2.35 (1.50–3.69)	13	7	3.00 (1.79–5.00)
<10th	38	15	4.32 (3.23–5.77)	46	15	5.28 (3.95–7.06)	37	14	4.16 (2.87–6.02)
>90th	17	12	2.40 (1.70–3.38)	13	13	1.82 (1.23–2.68)	17	13	2.07 (1.32–3.23)
**Ultrasound norms, percent** e									
<5th percentile	33	9	6.02 (4.41–8.23)	38	10	6.71 (4.94–9.13)	32	10	5.56 (3.77–8.21)
5th–<10th	10	8	2.16 (1.38–3.39)	11	7	2.71 (1.71–4.31)	9	7	2.22 (1.23–4.00)
10th–90th	45	78	Reference	44	77	Reference	47	77	Reference
>90th–95th	5	3	2.88 (1.52–5.45)	3	3	1.48 (0.67–3.28)	5	3	2.94 (1.40–6.16)
>95th	7	3	4.59 (2.68–7.86)	5	3	2.95 (1.62–5.36)	6	3	3.31 (1.62–6.77)
<10th	43	17	4.31 (3.27–5.68)	48	17	5.06 (3.83–6.69)	42	17	4.15 (2.91–5.90)
>90th	12	5	3.74 (2.44–5.73)	8	6	2.24 (1.37–3.65)	12	6	3.13 (1.83–5.35)
**Population norms, percent** f									
<5th percentile	9	3	3.00 (1.83–4.92)	11	4	3.60 (2.26–5.73)	13	3	4.06 (2.31–7.15)
5th–<10th	8	4	2.14 (1.29–3.54)	10	4	2.74 (1.69–4.46)	5	4	1.35 (0.65–2.81)
10th–90th	79	85	Reference	75	84	Reference	79	84	Reference
>90th–95th	2	4	0.62 (0.28–1.36)	<1	4	0.18 (0.04–0.75)	<1	4	0.12 (0.02–0.90)
>95th	2	4	0.50 (0.23–1.08)	4	5	0.93 (0.49–1.76)	3	5	0.73 (0.32–1.65)
<10th	17	7	2.53 (1.76–3.63)	21	7	3.15 (2.22–4.47)	18	7	2.62 (1.67–4.11)
>90th	4	8	0.56 (0.32–0.97)	4	8	0.60 (0.33–1.07)	4	9	0.46 (0.21–0.97)

Birth weight for GA at death (stillbirths) or delivery (live births) by the SCRN algorithm [17]. Stillbirths and live births in women with pregestational (type 1 or type 2) or gestational diabetes or with chronic hypertension or gestational hypertension/preeclampsia were excluded from the subset without maternal diabetes. Stillbirths and live births with malformations or chromosomal abnormalities or who were estimated to be <24 wk gestation at death or delivery were excluded from the non-anomalous subset. The subset with optimal estimation of GA is defined below. Percentages may add to slightly more or less than 100% because of rounding.

a In this subset, GA was estimated using an expected due date based on an ultrasound examination at ≤20 wk 6 d or last menstrual period that agreed with that ultrasound, and for stillbirths there was an interval of 7 d or fewer between the date the fetus was last recorded alive and the date fetal demise was first reported.

b Unadjusted OR for stillbirth for infants with birth weight in the percentile group shown compared to infants in the reference group from a logistic regression model that included effects for percentile group only.

The ORs adjusted for stillbirth risk factors as defined in Table 4, footnote b (excluding adjustment for pregestational hypertension and pregestational diabetes in the subset without maternal diabetes or hypertension), were similar and are not shown.

c Analysis weights that accounted for the basic study design plus other aspects of the sampling were used.

In the subset of pregnancies without maternal diabetes or hypertension/preeclampsia, unweighted sample sizes were 384 stillbirths and 1,402 live births. In the subset of non-anomalous singletons ≥24 wk gestation, unweighted sample sizes were 315 stillbirths and 1,661 live births. In the subset with optimalestimation of GA, unweighted sample sizes were 199 stillbirths and 1,226 live births.

d Individualized norm percentiles were derived using the fetal weight for GA equation from Bukowski et al. [15].

e Ultrasound norm percentiles were derived using the fetal weight for GA equation and standard error from Hadlock et al. [19].

f Alexander et al. population norm percentiles of birth weight for GA were used [18].

Simple linear interpolation was used with the Alexander et al. birth weight percentiles reported for completed weeks of GA in whole weeks to derive birth weight percentiles for GA in weeks and days.

LB, live birth; SB, stillbirth.

Among stillbirths identified as LGA by each of the norms, only one had a GA of more than 40 wk, and LGA was observed among preterm as well as term stillbirths. Hydrops was diagnosed among 11% (10/91), 14.5% (9/62), and 12.5% (3/24) of stillbirths identified as LGA using the individualized, ultrasound, and population norms, respectively. LGA was associated with stillbirth in the subset of non-anomalous pregnancies, which excluded the pregnancies with hydrops (Table 5).

Accounting for the time of death in stillbirths to determine fetal age did influence the proportion of SGA and LGA infants (Table 6). Increasing the accuracy of the fetal age estimate—from using GA at delivery to GA at delivery minus 2 d to GA at estimated time of fetal death—to determine weight-for-age percentiles decreased the proportion of stillbirths classified as SGA and increased the proportion classified as LGA using each of the three norms of fetal growth. ORs for stillbirth associated with SGA increased, and those associated with LGA decreased, when GA at delivery was used to determine birth weight percentiles for stillbirths instead of GA at death, regardless of which norms were used.

**Table 6:** Birth weight percentiles in relation to different GA estimates among singleton stillbirths.

Birth Weight Norms and Percentiles	Stillbirths			Live Births	Using Percentiles Based on GA at Delivery	
	GA at Death	GA at Delivery Minus 2 d	GA at Delivery		Crude OR for SB (95% CI)^a^	Adjusted OR for SB (95% CI)^b^
**Weighted sample size, number** c	528	561	570	1,382		
**Individualized norms, percent** d						
<5th percentile	33	44	48	9	11.25 (8.62–14.67)	11.27 (8.40–15.12)
5th–<10th	8	6	6	6	2.04 (1.33–3.14)	2.22 (1.36–3.62)
10th–90th	42	37	35	72	Reference	Reference
>90th–95th	4	3	2	5	0.87 (0.48–1.55)	1.02 (0.55–1.87)
>95th	13	11	8	7	2.48 (1.69–3.64)	2.15 (1.40–3.29)
<10th	41	50	54	15	7.34 (5.77–9.33)	7.70 (5.91–10.04)
>90th	17	14	11	12	1.79 (1.28–2.49)	1.68 (1.16–2.42)
**Ultrasound norms, percent** e						
<5th percentile	36	45	51	10	11.34 (8.79–14.63)	10.79 (8.11–14.35)
5th–<10th	9	8	7	7	2.17 (1.43–3.31)	2.24 (1.41–3.57)
10th–90th	43	38	34	77	Reference	Reference
>90th–95th	4	2	2	3	1.62 (0.83–3.18)	1.56 (0.76–3.19)
>95th	8	7	5	3	3.87 (2.37–6.34)	2.98 (1.73–5.12)
<10th	45	53	58	17	7.58 (6.01–9.57)	7.53 (5.80–9.78)
>90th	12	9	8	6	2.79 (1.86–4.19)	2.31 (1.48–3.61)
**Population norms, percent** f						
<5th percentile	12	23	25	4	8.98 (6.36–12.68)	9.20 (6.33–13.39)
5th–<10th	9	9	9	4	3.10 (2.06–4.66)	2.90 (1.78–4.72)
10th–90th	75	65	63	84	Reference	Reference
>90th–95th	2	1	<1	4	0.34 (0.13–0.89)	0.53 (0.19–1.45)
>95th	3	2	2	4	0.67 (0.36–1.25)	0.62 (0.34–1.15)
<10th	20	31	34	8	6.01 (4.59–7.88)	6.02 (4.44–8.16)
>90th	4	4	3	8	0.53 (0.31–0.89)	0.58 (0.34–1.01)

Birth weight percentiles for stillbirths ≥20 wk gestation using three GA estimates: GA at delivery, GA at delivery minus 2 d, and GA at death estimated using the SCRN algorithm [17]. Percentages may add to slightly more or less than 100% because of rounding.

a Unadjusted OR for stillbirth for infants with birth weight in the percentile group shown compared to infants in the reference group from a logistic regression model that included effects for percentile group only.

b Adjusted OR for stillbirth for infants with birth weight in the percentile group shown compared to infants in the reference group from a logistic regression model that in addition to the percentile group indicators included study site number; paternal age (<20, 20–34, 35–39, ≥40 y); the following maternal variables (categorized as shown in Table 1 or as noted): maternal age (<20, 20–34, 35–39, ≥40 y), race/ethnicity, education, marital status, insurance, family income, smoking during the 3 mo prior to pregnancy, alcohol use during the 3 mo prior to pregnancy, drug use, BMI, blood type, Rh factor, pregestational hypertension, pregestational diabetes, seizure disorder, and pregnancy history; and infant sex.

c Analysis weights that accounted for the basic study design plus other aspects of the sampling were used.

Unweighted sample sizes were 527, 561, and 570 stillbirths for GA at death, GA at delivery minus 2 d, and GA at delivery, respectively, and 1,821 live births. Unweighted (weighted) sample sizes included in computation of adjusted ORs were 491 (489) stillbirths and 1,665 (1,261) live births.

d Individualized norm percentiles were derived using the fetal weight for GA equation from Bukowski et al. [15].

e Ultrasound norm percentiles were derived using the fetal weight for GA equation and standard error from Hadlock et al. [19].

f Alexander et al. population norm percentiles of birth weight for GA were used [18].

Simple linear interpolation was used with the Alexander et al. birth weight percentiles reported for completed weeks of GA in whole weeks to derive birth weight percentiles for GA in weeks and days.

SGA and LGA birth weights based on ultrasound and individualized norm percentiles were significantly associated with an increased risk of preterm as well as term stillbirth. Using population norms, only SGA pregnancies were significantly associated with preterm and term stillbirth (Table 7). There was no significant difference in the GA distributions of stillbirths classified as LGA versus AGA or SGA using ultrasound or individualized norms.

**Table 7:** Birth weight percentiles among preterm and term stillbirths and live births.

Birth Weight Norms and Percentiles	Preterm SB and All LB			Term SB and LB		
	Preterm SB	All LB	Crude OR for Preterm SB (95% CI)^a^	Term SB	Term LB	Crude OR for Term SB (95% CI)^a^
**Weighted sample size, number** b	433	1,382		94	1,233	
**Individualized norms, percent** c						
<5th percentile	36	9	7.37 (5.52–9.84)	19	8	3.30 (1.80–6.04)
5th–<10th	7	6	1.88 (1.18–2.99)	12	6	2.83 (1.38–5.81)
10th–90th	41	72	Reference	49	73	Reference
>90th–95th	4	5	1.44 (0.85–2.45)	4	5	1.17 (0.45–3.07)
>95th	12	7	2.97 (2.03–4.35)	16	6	3.67 (1.97–6.84)
<10th	43	15	5.04 (3.88–6.54)	30	15	3.10 (1.86–5.15)
>90th	16	12	2.31 (1.67–3.21)	20	12	2.52 (1.45–4.39)
**Ultrasound norms, percent** d						
<5th percentile	39	10	7.30 (5.53–9.63)	21	10	3.08 (1.77–5.39)
5th–<10th	8	7	2.22 (1.44–3.45)	13	6	2.88 (1.42–5.86)
10th–90th	41	77	Reference	55	78	Reference
>90th–95th	5	3	2.96 (1.65–5.30)	2	3	0.92 (0.21–3.95)
>95th	8	3	4.49 (2.76–7.31)	9	3	4.69 (2.12–10.41)
<10th	47	17	5.22 (4.06–6.72)	34	16	3.00 (1.86–4.85)
>90th	12	6	3.76 (2.55–5.55)	11	6	2.74 (1.35–5.55)
**Population norms, percent** e						
<5th percentile	13	4	3.71 (2.47–5.55)	7	4	2.33 (1.00–5.41)
5th–<10th	8	4	2.25 (1.42–3.57)	12	4	3.68 (1.80–7.55)
10th–90th	76	84	Reference	69	84	Reference
>90th–95th	2	4	0.51 (0.23–1.17)	<1	4	0.29 (0.04–2.12)
>95th	1	4	0.31 (0.12–0.77)	11	5	2.81 (1.39–5.67)
<10th	21	8	2.97 (2.17–4.07)	19	8	3.02 (1.70–5.36)
>90th	3	8	0.40 (0.21–0.74)	12	9	1.67 (0.86–3.22)

Birth weight for GA at death (stillbirths) or delivery (live births) by the SCRN algorithm [17]. Percentages may add to slightly more or less than 100% because of rounding.

a Unadjusted OR for stillbirth for infants with birth weight in the percentile group shown compared to infants in the reference group from a logistic regression model that included effects for percentile group only.

b Analysis weights that accounted for the basic study design plus other aspects of the sampling were used.

In the subset used to assess risk of preterm stillbirth, unweighted sample sizes were 433 preterm stillbirths and 1,821 (preterm and term) live births. In the subset of term pregnancies, unweighted sample sizes were 94 stillbirths and 1,386 live births.

c Individualized norm percentiles were derived using the fetal weight for GA equation from Bukowski et al. [15].

d Ultrasound norm percentiles were derived using the fetal weight for GA equation and standard error from Hadlock et al. [19].

e Alexander et al. population norm percentiles of birth weight for GA were used [18].

Simple linear interpolation was used with the Alexander et al. birth weight percentiles reported for completed weeks of GA in whole weeks to derive birth weight percentiles for GA in weeks and days.

LB, live birth; SB, stillbirth.

## Discussion

This study demonstrates that stillbirth is associated with both growth restriction and excess growth. The extremes of SGA and LGA (<5th and >95th percentiles) were associated with the highest risk of stillbirth.

### Strengths

The strengths of this study lie in its geographically defined population-based design capturing live births and stillbirths, the large number of stillbirths evaluated, the accurate estimation of GA at death in stillbirths, the assessment of fetal growth using different fetal growth standards, and the ability to examine the contribution of factors affecting both birth weight and the risk of stillbirth. The systematic and standardized estimation of time of death and thus GA at death for stillbirths allowed for more accurate assessment of the association between birth weight and stillbirth and, consequently, of the association of stillbirth with LGA.

Prior studies have either not accounted for the interval between time of death and time of delivery of stillbirths [22]–[27] or have assigned an arbitrary interval of 2 d from GA at death to GA at delivery [28]–[30]. These approaches substantially overestimate GA at death [17],[31]–[33] and thus result in overestimation of the proportion of SGA infants and underestimation of the proportion of LGA infants.

Studies evaluating the interval between death and delivery have shown that in 25%–50% of stillbirths the interval was longer than 7 d [31]–[33]. Using the algorithm defined by the SCRN, over 40% of deaths were estimated to have occurred at least a week before delivery [17]. Thus, increasing the accuracy of the fetal age estimate in stillbirths, from GA at delivery to GA at delivery minus 2 d to GA at estimated time of death, decreased the proportion of stillbirths classified as SGA and increased the proportion classified as LGA.

Many prior studies of stillbirth have focused exclusively on the association of stillbirth with SGA [22]–[24],[28]–[30]. They compared risk of stillbirth in women with and without SGA infants, including LGA infants in the non-SGA comparison group. This approach resulted in failure to identify the association between LGA and stillbirth and also decreased the strength of the association of stillbirth with SGA. A retrospective study analyzed the association between both SGA and LGA and stillbirth, using population norms derived from the study population [27]. In that study, the proportion of SGA infants was 25% among stillbirths and 10% among live births, but the proportions of LGA infants were the same among stillbirths and live births, both 10%. That study was also limited by incomplete account of the confounding effects of stillbirth risk factors such as diabetes, maternal weight, hypertension, and smoking. Another study of the association between birth weight and risk of stillbirth found that birth weight in excess of 4,500 g was associated with increased risk of stillbirth [34]. However, infants with birth weight greater than 4,500 g are almost exclusively post-term and thus at risk of stillbirth due to prolonged duration of pregnancy [18],[19],[35].

A recent case series reported a higher than expected proportion of LGA among stillbirths. However, the majority of the LGA stillbirths in this series were related to fetal hydrops or maternal diabetes [36].

In our study, abnormal fetal growth was identified in twice as many stillbirths using ultrasound and individualized norms as when using population norms. Although SGA was associated with stillbirth based on all three norms, the association of stillbirth with LGA was observed only when using ultrasound or individualized norms. Differences in design may account for these results. The population norms by Alexander et al. are commonly used and were developed using birth weights from all pregnancies resulting in single live births, including those with complications associated with growth abnormalities, resulting in a wide range of birth weights between the 10th and 90th percentiles, classified as AGA [18]. In contrast, both the ultrasound and the individualized norms were derived from a cohort of uncomplicated pregnancies. Furthermore, the individualized norms account for maternal and pregnancy characteristics to the extent they affect birth weight in uncomplicated live born pregnancies, which may result in more accurate assessment of the expected fetal weight and deviations from expected size.

In a large population of uncomplicated pregnancies, population reference percentiles and, to a lesser degree, ultrasound norms were shown to overestimate the proportion of AGA and to underestimate the proportion of LGA infants [15]. Consistent with those findings, in this study only 8% of live births and 4% of stillbirths were classified as LGA by population norms. Using individualized norms, 12% of live births and 17% of stillbirths were classified as LGA. The underestimation of LGA and the high proportion of stillbirths classified as AGA by population norms may explain the observed lack of association between stillbirth and LGA based on population norms.

In individualized norms, the predictors of fetal growth, the sizes of their effects, and the ranges of their values were derived from a carefully selected population of almost 10,000 pregnancies without pregnancy or neonatal complications [15]. Among those predictors are ones with known association with SGA, such as smoking. This is likely because the relationship between smoking and fetal growth is complex. Smoking is associated with SGA, but the majority of those SGA pregnancies will not have adverse outcomes [37]. Smoking is also associated with a decreased risk of preeclampsia, a major risk factor for SGA [38]. Because of the opposing effects of smoking on birth weight, smoking was taken into account in individualization of fetal growth, despite its clear adverse effect on pregnancy outcome. Consistent with these observations, our sensitivity analysis showed that individualized norms with and without smoking, as well as with all the predictors, had similar patterns of association with stillbirth (Table 3). However, an argument might be made in general against accounting for maternal smoking in predicting optimal individualized expected birth weight, as smoking may have an adverse effect on a particular pregnancy, and the effect of smoking on an individual pregnancy is difficult to determine. The effect of smoking on fetal growth and adverse pregnancy outcome is complex because of its dual effect on both SGA and pregnancy complications due to a positive association of SGA directly with smoking, but a negative association indirectly through lower risk of preeclampsia. Additionally, the effect of smoking on SGA and complications of pregnancy is confounded by co-exposures and other characteristics, and is unclear with very low levels of exposure.

Consistent with prior studies, the findings of this study show that population norms are inferior to norms derived from uncomplicated populations, either ultrasound or individualized norms [39]. The ultrasound and individualized norms performed similarly in identifying pregnancies at risk of stillbirth. Both similarly detected SGA pregnancies associated with stillbirth in this study, and both, in a prior study, classified approximately 10% of uncomplicated pregnancies as SGA [15]. Ultrasound norms appear to underestimate the proportion of LGA pregnancies, both among stillbirth in this study and among uncomplicated pregnancies [15]. The association of fetal growth with other pregnancy outcomes, such as neonatal mortality and morbidity, was not investigated in this study. There is substantial uncertainty about the performance of different norms adjusting for fetal growth determinants [40],[41]. This study was not designed to compare customized norms. A previous study comparing customized and individualized norms showed that the latter performed better in identifying pregnancies with various complications [15]. An advantage of individualized norms is that the fetal growth predictors, their effect sizes, and the ranges of their values were derived from a large population of uncomplicated pregnancies rather than arbitrarily chosen.

The strength and pattern of the association between fetal growth and risk of stillbirth was similar in term and preterm pregnancies. SGA and LGA birth weights were associated with increased risk of stillbirth in preterm as well as term pregnancies using ultrasound or individualized norms. Using population norms, only SGA pregnancies had an increased risk of stillbirth, both preterm and term. The distribution of GA at death was similar among stillbirths classified as SGA, AGA, and LGA using ultrasound norms and also when classified using individualized norms, and the association of stillbirth with SGA and with LGA was observed when using both of these norms.

The association of LGA with stillbirth in this study was not related to post-term GA or known conditions that increase fetal weight and risk of stillbirth. Among stillbirths classified as LGA, only one pregnancy was greater than 40 wk. LGA was also associated with stillbirth among the subset of pregnancies that excluded hydrops and other congenital abnormalities. Because the relationship between congenital abnormalities and birth weight is complex and depends on the type of abnormality, we conducted analyses in pregnancies with and without congenital abnormalities. Both showed similar patterns of associations. Although the effect of maceration on a stillbirth's birth weight is uncertain, if birth weight is decreased in macerated stillbirths, this would decrease the strength of the association with LGA. However, the associations between SGA and LGA and risk of stillbirth were also observed in the subset with optimal estimates of fetal age that included non-macerated stillbirth infants with a short interval of less than 7 d between the time they were last reported alive and the time they were first identified as demised. Results of the subset analysis of pregnancies without pregestational or gestational diabetes or hypertension also showed similar associations with LGA.

Screening for gestational diabetes is performed in the US at 24 to 28 wk. Women at increased risk with a history of gestational diabetes, impaired glucose metabolism, or obesity are additionally screened in early pregnancy [42]. It is possible that a small proportion of women without those risk factors and delivering before routine screening was performed were not diagnosed, although those with LGA stillbirth are also recommended to be screened after stillbirth. However, it is unlikely that any misclassification would significantly affect the study findings, as the patterns of association between fetal growth and risk of stillbirth observed in the subset without pregestational or gestational diabetes and in the entire study population, including women with diabetes, were very similar.

### Limitations

A limitation of this study is that retrospective review of medical records was used to obtain birth weight, criteria for GA estimation, and certain maternal characteristics. However, these variables were recorded in medical records prospectively and thus were unlikely to be subject to substantial bias. Many of the characteristics used to determine individualized expected birth weight were missing and were replaced with reference values. However, individualized norms based on subsets of non-missing variables showed very similar findings (Table 3). In this study 70% of cases and 63% of controls agreed to participate. Analysis weights accounted for the observed differences between consenting and non-consenting women [16].

The mechanism of stillbirth in LGA pregnancies is not known. However, it has been suggested that LGA stillbirths may have relatively insufficiently large placentae, which, although not small per se, may be inadequate to support the metabolic demands of a large fetus, rendering it vulnerable to insults during pregnancy [36]. Burmeister et al. found that almost half of LGA stillbirths had placentae smaller than expected for fetal weight, and none larger than expected [36].

### Summary

In summary, when accounting for time of death and using norms developed in normal pregnancies, both SGA and LGA birth weights were associated with stillbirth in our study. The association is mainly related to severe SGA and LGA pregnancies, with birth weights either below the 5th or above the 95th percentile. Thus, classifying 10% of pregnancies as abnormally grown has the potential to identify 44%–46% of future stillbirths. This would provide an opportunity for prevention of stillbirth, especially in term pregnancies, when delivery is associated with relatively low neonatal mortality and morbidity. However, the effectiveness of stillbirth prevention would be expected to be decreased by inaccuracy of the fetal growth estimates. The association between large birth weights and stillbirth cannot be captured by population norms that include pregnancies with complications associated with growth abnormalities.

### Implications

Our results suggest that, contrary to current practices and recommendations, the most effective approach to identifying fetal growth abnormalities for prediction and prevention of stillbirth would focus on both severe SGA and LGA (<5th and >95th percentile) pregnancies and would use norms developed from normal pregnancies, rather than population norms, for fetal growth surveillance. This strategy would identify as at risk almost half of the pregnancies that would result in stillbirth. However, the majority of stillbirths would remain unidentified either because of inaccuracy of the fetal growth assessment or because they are not associated with growth abnormalities.

## Supporting Information

Table S1 **STROBE checklist.**
(DOC)

## Abbreviations

AGA: appropriate for gestational age
BMI: body mass index
GA: gestational age
LGA: large for gestational age
OR: odds ratio
SCRN: Stillbirth Collaborative Research Network
SGA: small for gestational age
